# *nanos*-Driven expression of *piggyBac* transposase induces mobilization of a synthetic autonomous transposon in the malaria vector mosquito, *Anopheles stephensi*

**DOI:** 10.1016/j.ibmb.2017.06.014

**Published:** 2017-07-01

**Authors:** Vanessa M. Macias, Alyssa J. Jimenez, Bianca Burini-Kojin, David Pledger, Nijole Jasinskiene, Celine Hien Phong, Karen Chu, Aniko Fazekas, Kelcie Martin, Osvaldo Marinotti, Anthony A. James

**Affiliations:** a Department of Molecular Biology and Biochemistry, University of California, Irvine, 3205 McGaugh Hall, Irvine, CA 92697-3900, United States; b Department of Microbiology and Molecular Genetics, B240 Med Sci Bldg., School of Medicine, University of California, Irvine, CA 92697-4025, United States

**Keywords:** Gene-drive, Genetically-engineered mosquitoes, Malaria vector

## Abstract

Transposons are a class of selfish DNA elements that can mobilize within a genome. If mobilization is accompanied by an increase in copy number (replicative transposition), the transposon may sweep through a population until it is fixed in all of its interbreeding members. This introgression has been proposed as the basis for drive systems to move genes with desirable phenotypes into target species. One such application would be to use them to move a gene conferring resistance to malaria parasites throughout a population of vector mosquitos. We assessed the feasibility of using the *piggyBac* transposon as a gene-drive mechanism to distribute anti-malarial transgenes in populations of the malaria vector, *Anopheles stephensi*. We designed synthetic gene constructs that express the *piggyBac* transposase in the female germline using the control DNA of the *An. stephensi nanos* orthologous gene linked to marker genes to monitor inheritance. Two remobilization events were observed with a frequency of one every 23 generations, a rate far below what would be useful to drive anti-pathogen transgenes into wild mosquito populations. We discuss the possibility of optimizing this system and the impetus to do so.

## 1. Introduction

Transposable elements have been proposed as a mechanistic basis for synthetic autonomous (self-mobilizing) gene-drive systems for introgressing anti-pathogen effector genes into wild mosquito populations (Kidwell and Ribeiro, 1992; Ribeiro and Kidwell, 1994; James, 2005). To function optimally, such a system must have the ability to remobilize (excise and integrate) itself and the desirable genes it is carrying from an initial insertion site to a new location in the genome of the target species. Furthermore, it should remobilize replicatively and with a frequency that allows it to reach fixation in a population within a useful timeframe (James, 2005). A *piggyBac* transposon construct lacking a source of transposase and integrated into the genome of the vector mosquito, *Anopheles stephensi*, could be remobilized to a new location by crossing the transgenic line with one expressing the transposase (O’Brochta et al., 2011). This ‘jumpstarter’ finding provided conceptual support for the development of synthetic elements capable of autonomous remobilization. We designed an autonomous gene-drive system based on the *piggyBac* element and the *nanos* gene 5′-and 3′-end flanking control DNA and tested it in transgenic *An. stephensi*. The construct was able to remobilize, however, at a frequency too low to be of practical use. Modifications including the replacement of control elements that promote a more robust expression of the *piggyBac* transposase, the use of alternative transposable elements, and the initial insertion of constructs at different locations on the mosquito genome may be able to increase remobilization efficiency.

## 2. Methods

### 2.1. Plasmids

The high-fidelity Phusion (Finnzymes, Wolburn, MA) DNA polymerase was used to amplify DNA fragments for plasmid construction. All fragments were amplified with oligonucleotide primers (Supplemental Table 1) designed with restriction sites for directional cloning into the shuttle vector *p*SLfa 1180fa (*p*SLfa; Horn and Wimmer, 2000).

#### 2.1.1. pBac3XP3-GFP[0.9nanos-pBacORF]

Specific primers were used to amplify *nanos* fragments from genomic *An. stephensi* DNA and the *piggyBac* open reading frame (ORF) from a *piggyBac* Helper plasmid (Handler et al., 1998). Amplification products were sub-cloned first into the Zero Blunt Topo vector (Invitrogen), then sequenced and sub-cloned into *p*SLfa with specific enzymes: *Hind*III/*Xba*I for the 900 base-pair (bp) promoter/5′-end untranslated region (UTR), *Xba*I/*BamH*I for the 1789bp *piggyBac* transposase ORF and *BamH*I/*EcoR*I for the *nanos* 3′UTR This cassette, [0.9nanos-*p*BacORF], was excised from pSLfa using *Asc*I and sub-cloned into pBac 3XP3-EGFPafm. The resulting vector, pBac3XP3-EGFP[0.9nanos-*p*BacORF] was used to generate the transgenic *An. stephensi* line As28+.

#### 2.1.2. pBacDsRed-attB[3.8nanos-pBacORF]

*The piggyBac* right inverted terminal repeat (ITR) and the 3XP3-EGFP-SV40 expression cassette in the *p*Bac3XP3-EGFP[0.9nanos-*p*BacORF] construct were replaced with the right ITR and the 3XP3-DsRed-SV40 expression cassette from pBac[3xP3-DsRedaf] (Nimmo et al., 2006) through the unique *Kas*I (vector backbone) and *Not*I (3′-end of SV40) sites in both constructs to produce the *p*Bac3XP3-DsRed-SV40[0.9nanos-*p*BacORF] plasmid.

The larger 3.8 *nanos* promoter sequence was synthesized *de novo* by Epoch Biolabs (Houston, TX) based on the genome sequence of the *An. stephensi nanos* gene (AY738090, ASTEI02887, Calvo et al., 2005), with *Xho*I and *Fse*I sites added to the 5′-end and a *Hind*III site added at the 3′-end. *Xho*I and *Fse*I were used to cut and clone the synthesis product into the pBluescript SK (−) plasmid to produce pBSK-Nan3.8. The larger promoter then was cut from the pBSK-Nan3.8 plasmid and used to replace the 0.9 *nanos* promoter in pBac3XP3-dsRed-SV40[0.9nanos-pBacORF] using the *Fse*I and *PpuM*I to produce pBac3XP3-DsRed-SV40[3.8nanos-*p*BacORF]. The *attB* sequence in *p*BattB[3xP3-DsRed2-SV40] (Labbé et al., 2010) was amplified using *attB* FOR and *attB* REV primers (Supplemental Table 1), which incorporate a *Pst*I site at each terminus of the product.

The DNA segment containing the *Pst*I site in the *DsRed* ORF was removed by digesting pBac[3xP3-DsRedaf] with *Sbf*I and *Not*I, blunting the cleaved termini with T4 DNA polymerase, and self-ligating the fragment containing the *piggyBac* transposon to produce pBac[3xP3-DsRedaf]SNKO, which contained a unique *Pst*I site in the right *piggyBac* ITR.

**Fig. 1:**
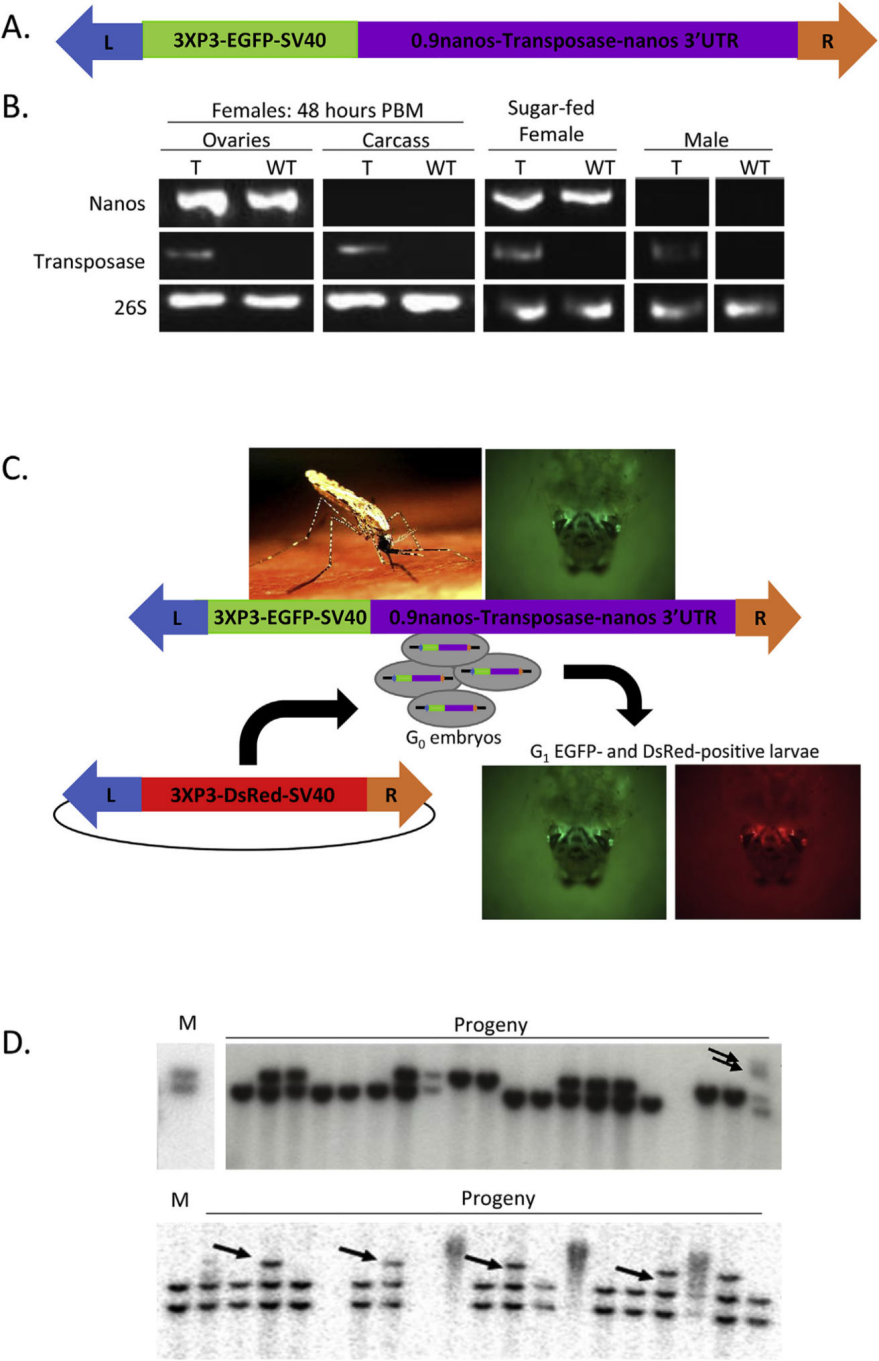
Endogenously-encoded transposase-mediated remobilization **A**) Schematic representation of the *piggyBac* construct used to engineer a transgenic *An. stephensi* line expressing transposase driven by *nanos* control elements. The construct encodes EGFP with a 3XP3 promoter driving expression in the eyes and the Simian Virus 40 3′UTR, as well as the *piggyBac* transposase coupled to 0.9 kb of genomic DNA immediately 5′ of the *nanos* coding region and the *nanos* 3′-end UTR. Both open reading frames are encoded between the *piggyBac* Left and Right ITRs (L, R). **B**) RT-PCR analysis of the presence of *nanos* gene transcript, *piggyBac* transposase transcript and 26 S ribosomal protein gene as a positive control in female ovaries and carcass and males. WT: wild type; T: transgenic As28+. **C**) Schematic of assay for integration of a non-autonomous element. A plasmid (thin black line) encoding the 3XP3-DsRed-SV40 transgene between *piggyBac* left and right ITRs (L, R) was injected without an exogenous transposase source into As28 + embryos (gray ovals) laid by transgenic EGFP-positive females. These females (image upper left) contain an integrated transgene comprising *piggyBac* left and right ITRs (L,R) flanking a 3XP3-EGFP-SV40 marker gene adjacent to the *piggyBac* transposase open reading frame (Transposase) flanked by the *nanos* 0.9 kb promoter, and 5′ - and 3′-end genomic DNA. This strain has EGFP fluorescence visible in the larval eyes (image upper right). As28 + embryos contain transposase expressed from the transgenic construct and if this expression results in functional transposase in the germline, the DsRed construct will be integrated into the *An. stephensi* genome, resulting in EGFP *and* DsRed expression in the eyes of larvae (images on lower right). **D**) Southern blot analysis of two mothers (M) and their progeny using a ^32^P-labelled probe for EGFP. Diagnostic DNA fragments present in progeny but not mothers are indicated with arrows and represent remobilization events.

The *attB* amplification product was cloned into pBac[3xP3-DsRedaf]SNKO at the *Pst*I site in the *piggyBac* right ITR to produce pBattB[3xP3-DsRedaf]SNKO. The orientation of the *attB* sequence was verified by gene amplification to ensure that two functional sets of *piggyBac* ITRs would be produced upon integration into the *attP* sequence present at the *An. stephensi* 44C line docking site (Isaacs et al., 2012), which was generated using pBac[3xP3-ECFPfa]-attP (Fig. 1, Nimmo et al., 2006). The 3XP3-DsRed-SV40 expression cassette was restored by cloning the 3XP3-DsRed-SV40 expression cassette from pBac[3xP3-DsRedaf] into pBattB[3xP3-DsRedaf]SNKO through the unique *Bst*BI and *Fse*I sites in each construct to produce pBattB[3xP3-DsRedaf]. The *nanos*-driven *piggyBac* transposase expression cassette from pBac[3XP3-DsRed-SV40] 3′Nan-pBORF-3.8Nan5′ was cloned into pBattB[3xP3-DsRedaf] through the unique *Sbf*I (in DsRed) and *Fse*I (at the 5′-end of 3.8 *nanos* promoter) sites in each construct to produce the *p*BacDsRed-attB[3.8nanos-*p*BacORF] plasmid.

### 2.2. Mosquito transformation

Transgenic *An. stephensi* carrying pBac3XP3-EGFP[0.9nanos-pBacORF] were created by injecting pre-blastoderm embryos with a mixture of pBac3XP3-EGFP[0.9nanos-*p*BacORF] (300 ng/µL) and *piggyBac* helper (200 ng/µL) plasmids using procedures described in Nirmala et al. (2006). A transgenic *An. stephensi* line carrying pBac3XP3-DsRed[3.8nanos-*p*BacORF] was obtained using site-specific integration. Embryos from the docking line 44C (Amenya et al., 2010; Isaacs et al., 2012) were microinjected with a mixture of pBac 3XP3-DsRed[3.8nanos-*p*BacORF] (300 ng/µL) plasmid and *φC31* integrase mRNA (400 ng/µL) as described (Nimmo et al., 2006).

### 2.3. Reverse transcriptase-PCR

Total RNA was isolated from whole animals and dissected tissues using RNAeasy Mini Kit (Qiagen) and treated with DNAse I (Invitrogen). The OneStep RT-PCR kit (Qiagen) was used for amplification of diagnostic products using primers listed in Supplemental Table 1. The reaction mixture was incubated at 50 °C for 30 min and 95 °C for 15 min. Amplification conditions were 94 °C for 1 min followed by 30 cycles of 94 °C for 1 min, 56 °C for 1 min and 72 °C for 1 min with a final extension for 10 min at 72 °C.

### 2.4. Southern blot analyses

Samples of genomic DNA from individual mosquitoes were isolated using Wizard Genomic DNA Purification kit (Promega) for Southern blot analyses and ~3.5 µg was digested using 30U of *EcoR*I or *Kpn*I in a 20 µL reaction. Digested DNA was resolved in a 0.8% agarose gel in Tris borate EDTA buffer (TBE: 0.089M Tris, 0.089M borate, 2 mM EDTA) at 70 V for 5 h or at 20 V overnight (~16 h). Gels were visualized after a 10-min stain in a GelRed 3,000X (Biotium), Tris acetate EDTA (TAE) solution (0.04M Tris, 0.04M acetate, 1 mM EDTA), soaked in a denaturation solution (1.5M NaCl, 0.5 NaOH) twice for 15 min, and soaked in a neutralization solution (1.5M NaCl, 0.5M Tris-HCL (pH 7.2), 1 mM EDTA) twice for 15 min. Gels were rinsed with deionized water. DNA transfer to a nylon membrane was set up according to standard protocols (Sambrook et al., 1989). Following transfer, nylon membrane blots were rinsed in 2X saline sodium citrate (SSC) and cross-linked at 1200 )µW/cm^2^ in a UV Stratalinker (Stratagene). To generate probes to detect insertions of the gene encoding the enhanced green fluorescent protein (EGFP) in the As28 + line, the first 450bp of the EGFP ORF were cloned into a TOPO TA vector (Invitrogen) generating the TOPO-EGFP plasmid, which then was digested with *EcoR*I. The resulting fragment containing the EGFP ORF DNA sequence was extracted and purified from an agarose gel.

Southern blot analysis also was used to identify G_17_ males from the 3.8nanos-attP44c colony that had one copy of each of the genes encoding the enhanced cyan fluorescent protein (ECFP) and *Discosoma species* Red (DsRed) marked constructs. Genomic DNA isolated from individual males was divided such that one-half was digested with *Kpn*I and probed with ^32^P-labelled ECFP DNA probe, and the remainder digested with *EcoR*I and probed with the ^32^P-labelled DsRed DNA probe. Labelled probes targeting *ECFP* or *DsRed* genes were generated by amplification of 600–700 bp fragments from plasmids bearing the marker genes (primers listed in Supplemental Table 1).

The amplification or digestion products were gel-electrophoresed, extracted and amplified with ^32^P-labelled dATP and dCTP (Perkin-Elmer) using Megaprime DNA labeling system (Amersham).

### 2.5. Splinkerette PCR

Splinkerette PCR was performed as described previously (Potter and Luo, 2010). Genomic DNA was extracted from adult mosquitoes using DNeasy Blood & Tissue (Qiagen) or Wizard Genomic DNA Purification kits (Promega) and digested with *Bst*I. Amplification products were resolved in agarose gels; two fragments were amplified from the dual reporter construct at the 44C site with the piggyBac5Rev primer and were ~350 and ~400bp in length, respectively. Any fragment of any other size was considered diagnostic for remobilization. All fragments were gel-extracted, purified and sequenced. Diagnostic amplifications to identify the genotype of mosquitoes derived from the dual-reporter line with exceptional phenotypes were performed with primer combinations as depicted in Fig. 3 with primers listed in Supplemental Table 1.

### 2.6. Inverse PCR

Inverse PCR was performed as described previously (Handler et al., 1998) with primers listed in Supplemental Table 1. Inverse PCR and splinkerette PCR are analogous procedures that provide the same information, however we found that splinkerette PCR was consistently effective for identifying the DNA flanking the *piggyBac* left ITR, whereas Inverse PCR was more reliable for identifying the DNA flanking the *piggyBac* right ITR. The Inverse PCR protocol was performed initially to identify individual mosquitoes that had been observed to have only one fragment for each *ECFP* and *DsRed* genes after probing of the genomic DNA by Southern blot. Following screening for exceptional phenotypes, genomic DNA was extracted from individual mosquitoes and inverse PCR performed on pools of genomic DNA aliquots from individuals of the same phenotype. Digests were performed with either *HeaI*II or *Msp*I for two hours and purified by ethanol precipitation. Ligations were performed using T4 DNA ligase (New England Biolabs) with a total reaction volume of 400 µL, overnight at 4 °C. DNA from ligation reactions was purified by ethanol precipitation and used as a template for gene amplification with primers listed in Supplemental Table 1. Amplification products were run on agarose gels and diagnostic fragments extracted using QiaQuik Gel Extraction kit (Qiagen), cloned into pSC-B-amp/kan using StrataClone Blunt PCR Cloning kit (Agilent Technologies) and transformed into Mix & Go Competent *Escherichia coli* strain JM109 (Zymo). Resulting bacterial colonies were picked and grown for plasmid amplification, and DNA isolated using Zyppy Plasmid Miniprep kit (Zymo) and sequenced by Laguna Scientific, a local fee-for-service company.

## 3. Results

### 3.1. A synthetic autonomous transposon; mobilization detected by Southern blot analyses

As a first step towards generating a synthetic autonomous construct, the *piggyBac* transposon was used as the basis for a transgene cassette that encoded *EGFP* as a marker gene and the *piggyBac* transposase under the control of the *An. stephensi nanos* promoter and 5′- and 3′ -end UTR (Fig. 1A). Work in the fruit fly, *Drosophila melanogaster*, showed that the *nanos* promoter and 5′ -end UTR drive expression of its mRNA in the maternal follicle cells and the 3′-end UTR mediates localization of the transcripts to the germline (Ali et al., 2010; Chen and McKearin, 2003; Curtis et al., 1995; Doren et al., 1998; Gavis et al., 1996; Gottlieb, 1992; Tracey et al., 2000). Orthologous mosquito genes appear to share similar expression characteristics (Calvo et al., 2005; Adelman et al., 2007; Meredith et al., 2013). Following transformation of *An. stephensi*, a line designated As28 + with four separate copies of the integrated construct was chosen for further analyses (Jimenez, 2009). RT-PCR analysis revealed that unlike the products of the endogenous *nanos* gene, the recombinant transposase was expressed in all tissues assayed including female somatic and male tissues (Fig. 1B). To assay this line for transposase activity, embryos were injected with an additional construct conferring *DsRed* expression in the eyes. The *DsRed* gene was flanked by the *piggyBac* left and right ITRs, but no transposase was encoded on the construct and no helper was introduced during the microinjection (Fig. 1C). Integration of this second construct was seen at a rate (4 insertions from 430 embryos injected, 0.93%) comparable to that observed with helper plasmid injection (17 insertions from 1000 embryo injections,1.7%) showing that the transposase expressed from the As28 + transgenic mosquitoes was active in the germline and so we expected that the construct would be able to self-mobilize.

A series of Southern blot analyses were performed on genomic DNA collected from individually outcrossed transgenic mothers and their offspring to assess whether any new insertions of the construct could be identified in progeny that were not present in the mother. Such insertions would be apparent by additional diagnostic DNA fragments or fragments of differing size in Southern blot analyses, and would be suggestive of transposase-mediated remobilization events. Two such events were detected in 386 progeny originating from 21 mothers (Fig. 1D). As each of the progeny samples results from a single gamete from each mother, we can calculate a remobilization frequency of 0.52% or 0.0052 remobilizations per gamete in this assay.

### 3.2. A dual-marker transgenic line, 3.8nanos-attP44C, for visual detection of remobilization

**Fig. 2:**
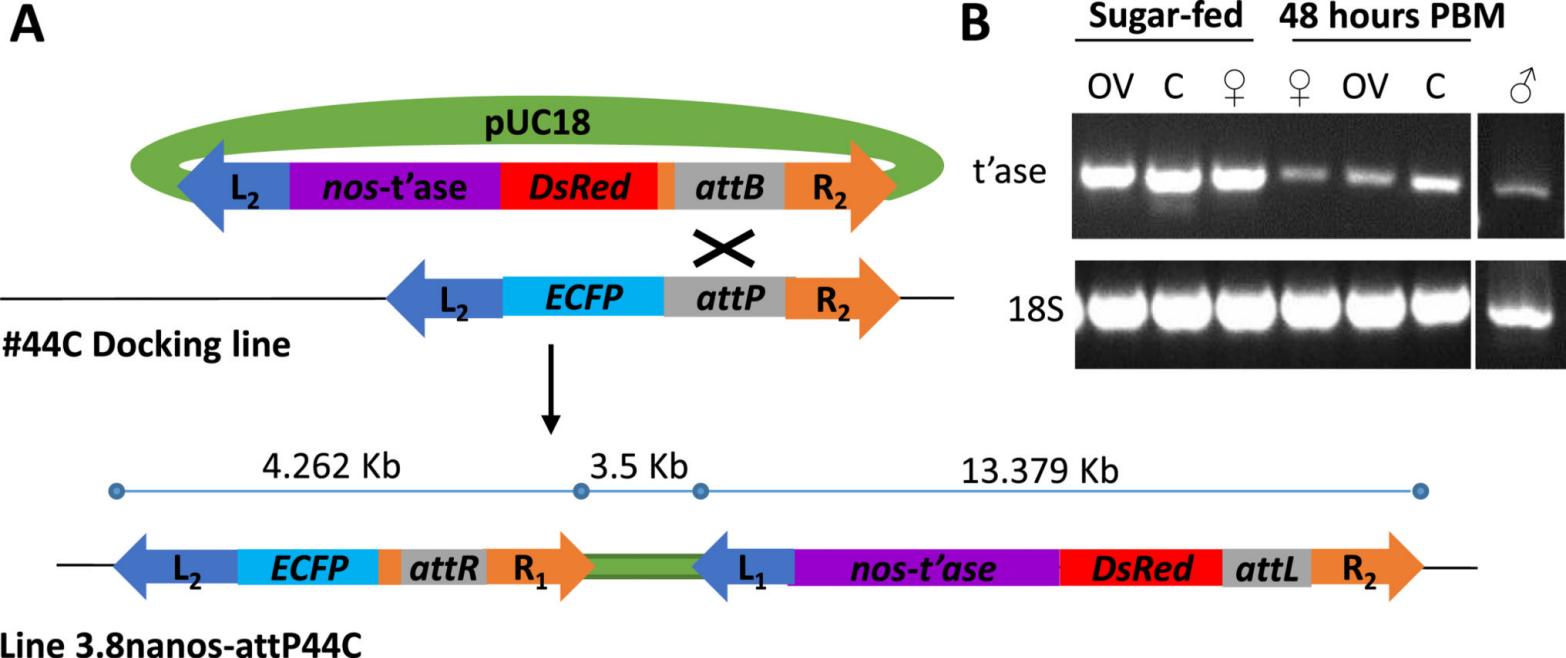
Generation and expression characterization of 3.8nanos-attP44C, a transgenic *An. stephensi* dual-reporter for transposon mobilization **A**) Schematic representation of the transgenic design. *DsRed*, the *piggyBac* transposase open reading flanked by 3.8 kb of the 5′ region of *An. stephensi nanos* and the *nanos* 3′untranslated region (nos-t’ase) and a *φC31 attB* site were encoded between *piggyBac* ITRs and the plasmid was used to transform attP44C line with *φC31* integrase to generate two tightly-linked markers. **B**) RT-PCR analysis of transposase expression in 3.8nanos-attP44C in sugar-fed (never blood-fed), 48 h post-blood meal (PBM) females (OV: ovary; C: carcass) and males.

**Fig. 3:**
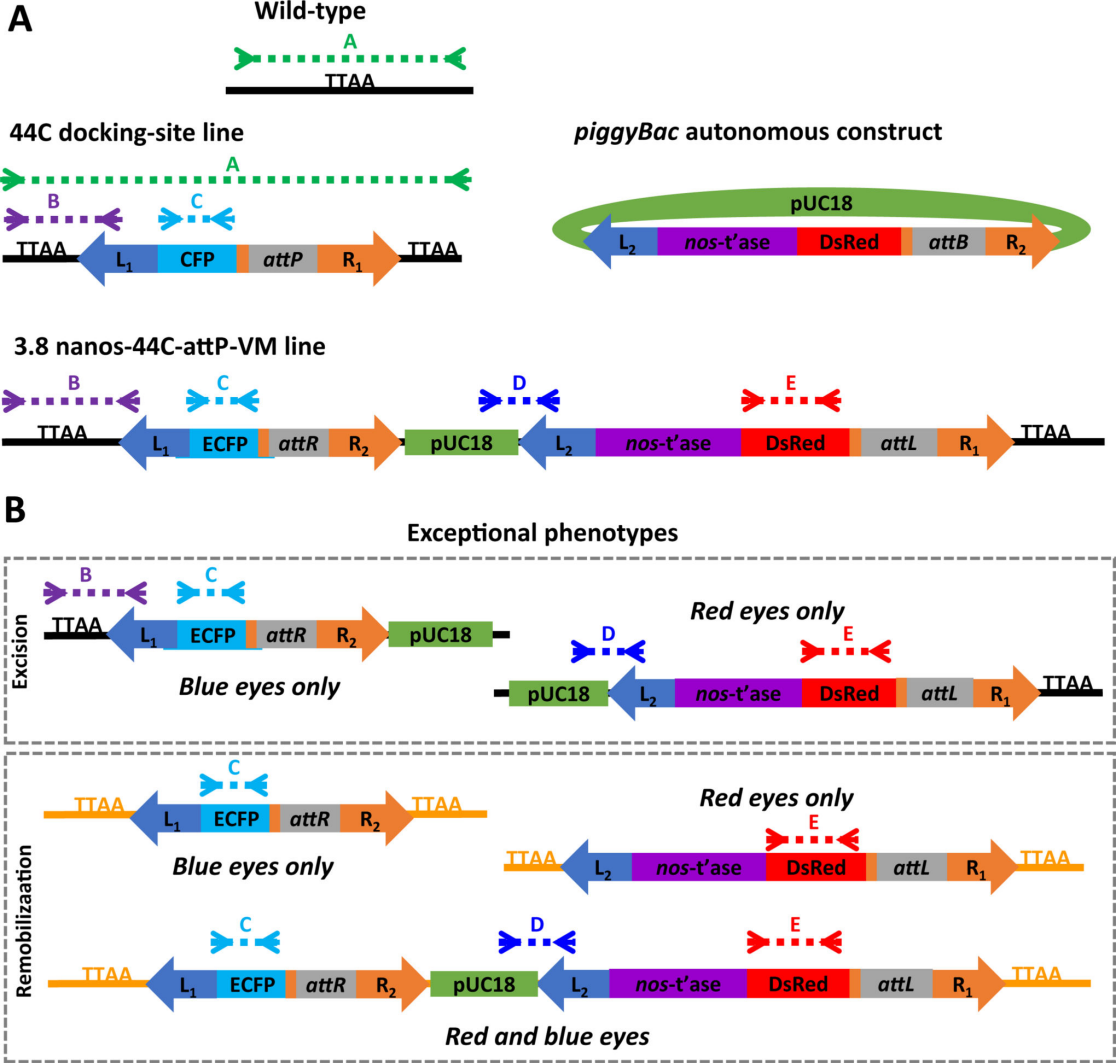
Schematic representations of gene structures and expected amplification products resulting from autonomous mobility of transgenes **A**) The 44C docking site line comprises a transgene consisting of the left (L_1_) and right (R_1_) inverted terminal repeats of the *piggyBac* transposable element flanking the coding sequence of the enhanced cyan fluorescent protein (ECFP) driven by a 3XP3 promoter and sequences for site-specific recombination (*attP*) from the phage *φC31* (Isaacs et al., 2012) integrated into the genome (horizontal black line). *piggyBac* transposase-mediated insertion causes a target-site duplication (TTAA) flanking the transgene. *φC31* recombinase-mediated integration of a *piggyBac* autonomous construct comprising a cloning plasmid (pUC18) carrying an additional set of *piggyBac* left (L_2_) and right (R_2_) inverted terminal repeats, the *piggyBac* transposase coding sequence driven by the *nanos* promoter (*nos*-t’ase), the DsRed fluorescent protein coding sequence (DsRed) driven by a 3XP3 promoter and the *φC31 attB* (*attB*) site yields the 3.8 nanos-attP44C VM line. DNA derived from wild-type and 44C docking-site line mosquitoes generates diagnostic amplification fragments of 384 and 4396 bp, respectively, when using primer set A. Similarly, primer sets B and C yield fragments of 404 and 654 bp, respectively, when DNA samples from 44C docking-site and 3.8 nanos-attP44C mosquitoes are used as templates. Primer sets D and E are specific to the 3.8 nanos-attP44C line and produce fragments of 182 and 621 bp, respectively. **B**) The generation of exceptional phenotypes (Blue or Red eyes only) can result from excision or remobilization of portions of the 3.8 nanos-attP44C transgene complex. Primer sets B, C, D and E are diagnostic for excision events in the combinations illustrated. Remobilization puts portions of or the entire transgene complex at a new site in the genome (horizontal orange line). Inverse PCR techniques are used to identify the new genomic location.

A new remobilization construct was generated with a number of improved characteristics. First, the final construct included two marker genes, *ECFP* and *DsRed*, each flanked by a set of *piggyBac* ITRs, such that the marker genes were linked tightly and could be remobilized either jointly or separately (Fig. 2A). Excision and remobilization events segregating the two fluorescent markers could be identified by visual screening of the eyes allowing the selection of individuals with relevant phenotypes (only one of the markers expressed in the eyes) for further molecular characterization. Second, the amount of 5′ flanking DNA from the putative *nanos* gene promoter was increased from 0.9 to 3.8 kb based on the previous observation that the former was not sufficient to specify tissue- and sex-specific expression characteristics. DNA fragments of ~1.5 kb comprising the putative promoter and 5′-end sequences of the *Ae. aegypti* and *An. gambiae nanos* orthologs were shown previously to be sufficient to drive abundant sex- and tissue-specific expression (Adelman et al., 2007; Meredith et al., 2013). However, following injection and recovery of transgenic mosquitoes, RT-PCR analysis of total RNA collected from sugar-fed and blood-fed whole females, dissected female tissues and whole males showed *piggyBac transposase* transcript present in all samples assayed (Fig. 2B). Third, a transformation scheme was devised to integrate a single copy of the gene-drive construct using *φC31*-mediated recombination to place the construct at a known and characterized location in the genome (Fig. 3). The transgenic line bearing the construct was generated by microinjection of the *An. stephensi* docking site line 44C (Amenya et al., 2010; Isaacs et al., 2012). G_1_ individuals with both DsRed- and ECFP-fluorescent eyes were scored as positive for integration of the construct into the 44C site. A line, 3.8nanos-attP44C, was derived from intercrossing these individuals and was maintained initially with screening at every generation.

The fluorescent marker arrangement allowed us to separate individuals with evidence of putative movements during larval screening by identifying those with the exceptional phenotypes of only ECFP or only DsRed fluorescence in the eyes (Fig. 3). Genomic DNA samples from exceptional individuals were analyzed by gene amplification to verify the observed phenotype and to characterize remobilization events. This diagnostic gene amplification scheme allowed us to characterize individuals as excision or remobilization events since an exceptional phenotype could arise by excision of the other marker gene, or a remobilization of a marker gene to a new genomic location and segregation from an occupied ECFP-marked 44C docking site.

**Fig. 4:**
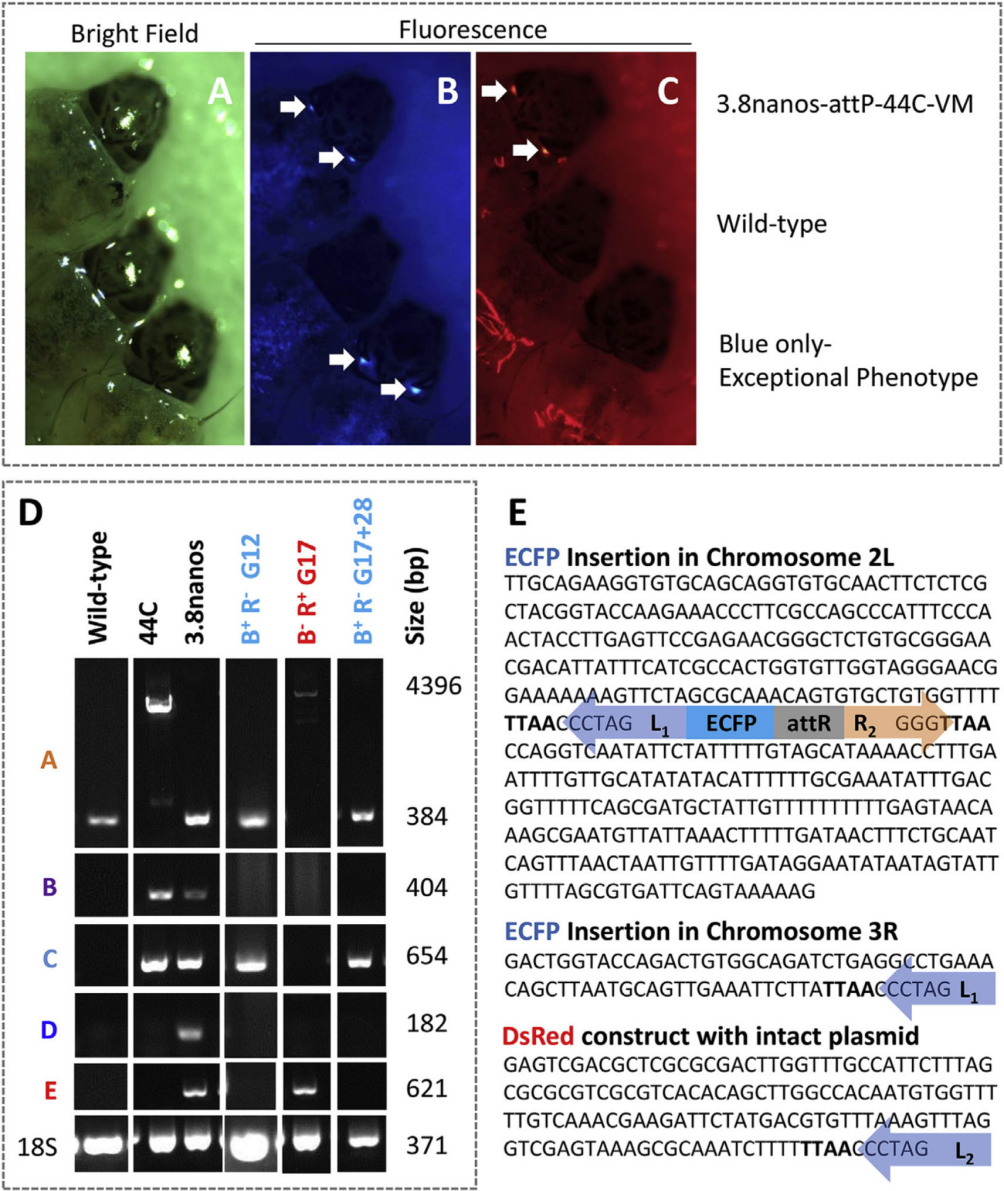
ECFP construct mobilization Mosquitoes of the 3.8nanos-attP44C and -VM lines were screened for ECFP and DsRed in the eyes (white arrows). From top to bottom in each picture: 3.8nanos-attP44C-VM larvae with both fluorescent markers, a larva from the wild type colony, a 3.8nanos-attP44C-VM larvae with an exceptional phenotype (ECFP, but not DsRed). **A**) Bright field microscopy **B**) ECFP filter **C**) DsRed filter **D**) DNA derived from wild-type, 44C docking site line and 3.8 nanos-attp44cVM line mosquitoes, and exceptional individuals amplified with the designated primer sets (Fig. 3) produces amplicons supporting remobilization of the ECFP portion of the transgene complex. **E**) DNA sequence flanking the *piggyBac* left and right ITRs identified by inverse PCR in exceptional individuals. Abbreviations: *attB, attL, attP* and *attR, φC31* attachment sites bacteria, left, phage and right, respectively; ECFP, enhanced cyan fluorescent protein; DsRed, *Discosoma* sp. red fluorescent protein; L_1,2_, R_1,2_, left and right *piggyBac* inverted terminal repeats, respectively; nos-t’ase, *nanos* promoter and *piggyBac* transposase open-reading frame; TTAA, *piggyBac* transposon recognition tetranucleotide.

A number of individuals with exceptional phenotypes were identified immediately following line establishment and we suspected some of these to have resulted from expression of the transposase from the injected plasmid. *φC31*-mediated insertion at the 44C site was validated by gene amplification and DNA sequencing, confirming the desired dual-marker construct illustrated in Fig. 3. However, it is possible that the *piggyBac* transposase encoded in *p*BacDsRed-attB[3.8nanos-*p*BacORF] was expressed during the initial injection and it could have mediated additional insertions at other genomic locations. An ECFP-only individual was identified in generation 12 (G_12_) and analysis of its DNA by gene amplification profiling and splinkerette PCR showed that the *ECFP* construct was at a genomic location other than the 44C integration site (Fig. 4A – D). The integration was confirmed by sequencing to be on chromosome 3R at a TTAA site on scaffold location 1480193–14 80196 (Fig. 4E; Indian Strain, VectorBase.org). Additionally, a G_17_ DsRed-only individual was characterized by gene amplification and inverse PCR (analogous to splinkerette PCR) to be the result of an excision of the *ECFP* construct (Fig. 4D and E).

**Fig. 5:**
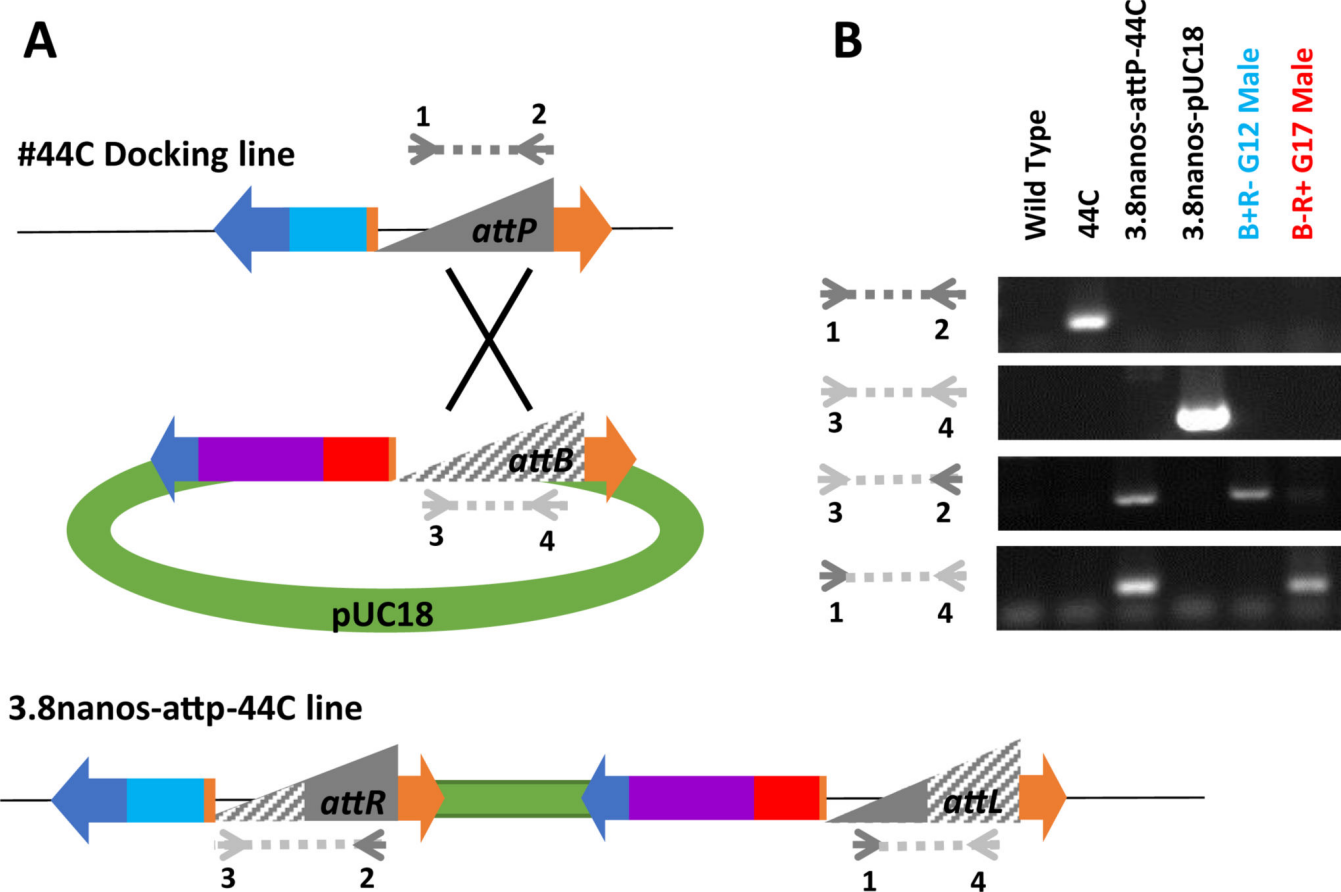
Verification of mobilization out of the 44C genomic location **A**) Gene amplification scheme to differentiate the presence of a *φC31 attP* site present in the docking line and *attB* site present on the injected plasmid from *attR* and attL sites. Gene-specific primers are shown as arrowheads flanking a diagnostic fragments represented as a dotted line. **B**) PCR analysis on individuals with exceptional phenotypes, ECFP- or DsRed-only, collected at generation 12 (G12) and generation 17 (G17) respectively. Wild-type *An. stephensi* were used as a negative control and ECFP-only mosquitoes from docking line 44C and ECFP/DsRed mosquitoes from line 3.8nanos attP44C were used as positive controls for *attP* and *attR* sites respectively. The injected plasmid *p*BacDsRed-attB[3.8nanos-*p*BacORF] was used as a positive control for the *attB* site.

To confirm that the *ECFP* integration on chromosome 3R was mediated by transposition out of the 44C docking site following its prior *φC31*-mediated integration and not an insertion mediated by transposase expressed during injection of G_0_ embryos, a set of gene amplification primers were designed to differentiate the *attR* site that would be present on the *ECFP* construct in the former from the *attB* site that would be present in the latter (Fig. 5A). Gene amplification analysis identified an *attR* site and not an *attP* site in the DNA of the ECFP-only individual, confirming this event as a remobilization event (Fig. 5B). Similarly, gene amplification showed the presence of an *attL* site in the genomic DNA of the G_17_ DsRed-only individual leaving the *DsRed* construct behind, indicating that the *ECFP* portion of the construct was mobilized out of the site (Fig. 5B). We can conclude from these data that remobilization occurred at least once by generation 17.

### 3.3. Identification and characterization of remobilization in 3.8nanos-attP44C-VM

In order to ensure that individuals identified as only ECFP-positive or only DsRed-positive in future generations represented transposase activity originating from expression of the transgene located at the 44C site, it was necessary to isolate the *φC31* integrase-mediated site-specific integration from genotypes resulting from G_0_ transposase-mediated remobilization and subsequent mobilizations being maintained in the colonies. Eight G_17_ males from 3.8nanos-attP44C were outcrossed individually and then assayed molecularly for their genotype. Male #3 was found to have only one copy of *ECFP* and *DsRed* by Southern blot analysis and inverse PCR showed that they were both present at the 44C site as originally intended (Supplemental Fig. 1). Progeny of male #3 were used to found the line 3.8nanos-attP44C-VM. Generation number will be counted as a continuation of line 3.8nanos-attP44C, so that the founding generation of 3.8nanos-attP44C-VM is G_17_.

To identify remobilization events, 3.8nanos-attP44C-VM males were outcrossed at every generation from G_17_ for five generations and the progeny screened for exceptional phenotypes, which include not only mosquitoes showing only ECFP or DsRed, but also ECFP- and DsRed-positive male individuals. The 44C site is located on the X-chromosome, so by outcrossing males, we can identify any male progeny with fluorescence as exceptional, since the original insertion should only be passed to females. After five generations of outcrossing, no exceptional phenotypes were seen. We reasoned that the mobilization frequency could be influenced by transposase dose, so we began maintaining the line by intercrossing. Larvae were screened at every generation for five additional generations without identification of exceptional phenotypes.

After 28 generations, four G_45_ males from the transgenic line were outcrossed and 38 out of 449 total progeny were ECFP-only individuals (8.4%, Fig. 4A, B and C). In order to identify how many remobilization events had occurred over the past generations 10 ECFP-only males were outcrossed to generate individual families to increase the amount of genomic DNA available for analysis.

Only one remobilization event was captured; a remobilization of the *ECFP*-marked portion of the construct was identified by inverse PCR to be on chromosome 2 L, scaffold_00100 (Indian Strain, VectorBase.org, Fig. 4E) at the TTAA sequence at position 272403–272406. TTAA sequences flanking the construct support the conclusion that the construct was precisely excised and reintegrated by *piggyBac* transposase (Fraser et al., 1996).

## 4. Discussion

We report here on the activity in *An. stephensi* of transposon-based synthetic autonomous gene drive constructs, As28 + and 3.8nanos-attP44C, encoding *piggyBac* transposase under the control of the *An. stephensi nanos* gene promoter, 5′- and 3′-end UTRs. The recovery of these constructs at genomic loci other than those of their original insertion sites is a proof-of-principle that a synthetic transposon construct can be designed to self-mobilize. We observed two mobilization events in the As28 + line in 21 mother-progeny sets and this represents an estimated remobilization frequency of 0.0052/gamete. In the 3.8nanos-attP44C lines, we detected two events over 45 generations at what must be a much lower frequency per gamete. Thus, it appears that remobilization occurred at a higher rate in the As28 + line than in the 3.8nanos-attP44C lines. An alternative mechanism of relocation by recombination was ruled out in the exceptional individuals recovered from 3.8nanos-attP44C because target site (TTAA) duplications were detected flanking the right and left ITRs of the remobilized construct, supporting the conclusion that mobilization occurred by a cut-and-paste mechanism into the TTAA target site typical for *piggyBac* insertions (Fraser et al., 1996). We did not observe mobilization of the *DsRed*-marked construct in the 3.8nanos-attP44C transgenic lines. While the overall number of mobilizations observed is too small to be conclusive, it may be that this construct was prohibitively large. However, the *piggyBac* transposase has been demonstrated in other systems to faithfully remobilize a lengthy cargo, up to 100 kb (Li et al., 2011; Ding et al., 2005). It will be important to demonstrate that a transposon-based gene drive will be able to accommodate cargo in mosquitoes in order to drive anti-pathogen effector molecules into target populations.

The difference in mobilization frequencies between the two transgenic lines is puzzling and likely reflects a contribution of several possible factors. Perhaps some events were not captured in the 3.8nanos-attP44C lines due to mosquito line maintenance requirements. For example, a rare event may not persist in the colony for more than one generation since only a proportion of total mosquito embryos produced each generation are used to maintain the colony. In the As28 + line, mother progeny sets were analyzed, so detection did not necessitate the mobilization being frequent in the colony.

Going forward with the development of a usable *piggyBac*-based gene-drive in *An. stephensi*, it will be important to identify the contribution of transposase dose and genomic context to construct mobility. The As28 + transgenic line contained four transposase gene copies, whereas the 3.8nanos-attP44C lines had only one, and increased transposase abundance could explain the higher frequency of construct self-mobilization. However, the *piggyBac* element does not mobilize in *Ae aegypti*, even in the presence of functional transposase (Palavesam et al., 2013; Sethuraman et al., 2007). Furthermore, *piggyBac* element mobilization in *D. melanogaster* has been attributed to genomic context, not transposase abundance, while no genomic context has been identified that supports remobilization in *Ae. aegypti* (Esnault et al., 2011; Palavesam et al., 2013; Sethuraman et al., 2007).

The assay in which a plasmid encoding a DsRed marker gene between the *piggyBac* left and right ITRs was injected without a transposase source into the As28 + transgenic line resulted in efficient integration of the marked construct, showing that functional transposase is expressed from the integrated transgene and is capable of mediating integration at a frequency comparable to that of the injected helper plasmid in the original line generation. This integration frequency (0.97%) and the remobilization frequency observed in this line (0.52%) also are within the same order of magnitude. If integration of a *piggyBac* construct using a similar assay in the 3.8nanos-attP44C line is higher than the observed remobilization rate, we would suspect either transposon regulation at the level of genome integration or a difference in the make-up of the construct instead of a difference due to transposase dose. This could be explored by insertion of the constructs into alternative genomic locations that may support a more robust expression of the transposase; a number of *An. stephensi* lines bearing *attP* sites at different locations are available that could be used to test this (Amenya et al., 2010; N. Jasinkiene, personal communication).

Heritable *piggyBac* construct mobilization may be influenced by the amount of available transposase in the germline tissue. We observed that, unlike the *Ae. aegypti* and *An. gambiae nanos* control elements (Adelman et al., 2007; Meredith et al., 2013), the nucleotides up to nearly 4 kb to the 5′-end of the transcription start site of *An. stephensi nanos* were not sufficient to drive tissue-specific expression of the transposase, but induced expression in both germline and somatic female tissue and in males. A more restricted promoter that drives accumulation of *piggyBac* transposase to a high level in the ovaries may increase germline transposition frequency.

Synthetic, non-autonomous *piggyBac* constructs can be mobilized in the *An. stephensi* genome using a ‘jumpstarter’ helper line expressing the *piggyBac* transpose (O’Brochta et al., 2011). This enhancer-trap mechanism supported the hypothesis that a *piggy-Bac* transposase expressed from the *An. stephensi* genome could remobilize synthetic constructs in the genome to a new location, and a synthetic construct comprising a *piggyBac* transposase gene cloned between the *piggyBac* left and right ITRs in theory could remobilize itself. Our data validate that this is possible. However, remobilization frequencies in all lines are far below the 10% transposition per insert per generation that is predicted by models to be necessary for a useful gene-drive mechanism (Ribeiro and Kidwell, 1994; Rasgon and Gould, 2005; Marshall, 2008).

Crossing our lines with a helper line reported by O’Brochta et al. (2011) may provide additional insight into why mobility in our line is so low. Furthermore, while transposase transcript and protein were not quantified in the helper lines, such analyses may give an insight into the contribution of transposase dose to construct mobility.

It is also possible that the transposase is being post-transcriptionally regulated by the Piwi-interacting RNA (piRNA) pathway. piRNAs are derived from regions of the genome that have remnants of transposons to which the species was exposed previously in its evolutionary history. It may be that an ancestor of our *An. stephensi* line encountered one or more members of the *piggyBac* transposon family. A BLAST of the currently available assembled and annotated *An. stephensi* genomes identifies regions 19 to 40 nucleotides in length with 86–100% similarity to *piggyBac* transposase (VectorBase.org), but data are not currently available as to whether these regions are piRNA producers. These results are consistent with data from other anopheline species that contain regions in their genomes with similarity to *piggyBac* transposase (Fernandez-Medina et al., 2011; Marinotti et al., 2013). It could be that ancient exposures to ancestral *piggyBac* transposons provide enough sequence to establish repression of the contemporary transposon. If indeed piRNA regulation was influencing transposase availability, the construct can be redesigned to avoid this regulation by altering the nucleotide sequence. Since the regulation is mediated by nucleotide sequence specificity, if the transposase is encoded with different codons, the transposase transcript would essentially be invisible to the piRNA machinery.

In addition to modifications to improve tissue specificity, optimize transposon dose and genomic context and to avoid post-transcriptional regulation, alterations to the encoded transposase itself may improve construct mobility. A hyperactive *piggyBac* transposase has been reported to increase transposition nine-fold in mammalian cells (Yusa et al., 2011); it would be interesting to see whether expression of this transposase from our construct would yield a more efficient transposase-based genetic drive.

The *piggyBac* element has been the most successful transposon-based platform so far for genetic applications in *Anopheles species*, but it is possible that another transposable element would be more useful. Transposons have been sought from other organisms that have mobility in *Anopheles* species and *Ae. aegypti* with the hope of applying them as tools for genetic manipulation, including gene-drive and molecular genetic studies. However, specific transposable elements do not exhibit the same mobility characteristics in mosquitoes as they do in *D. melanogaster* and other insect species (O’Brochta et al., 2003; Wilson et al., 2003; Palavesam et al., 2013; Scali et al, 2007; Sethuraman et al, 2007). For example, P elements can mediate transposition of a transgene into the *D. melanogaster* genome, but are not effective for transformation of any mosquito species. The *Minos* element, which is remobilized following integration into the *D. melanogaster* genome in both somatic and germline tissues, moves only in the soma in *An. stephensi* (Scali et al, 2007). A *Herves* element isolated from *An. gambiae*, and later identified in wild populations of this species as well as *An. merus* and *An. arabiensis*, appears to be active in *An. gambiae* (Arensburger et al, 2005). An autonomous construct based on it could be tested for mobility in *An. stephensi* and *An. gambiae*.

It is possible that inundative releases of transgenic mosquitoes may be sufficient for the goal of fixation of an anti-pathogen genotype into wild populations, but the predicted benefits afforded by gene-drive technology in terms of reduced cost and effort of implementation over an inundative release strategy, makes developing the technology a worthwhile pursuit (Macias and James, 2015). Synthetic gene-drive systems based on homing endonucleases, MEDEA and on CRISPR/Cas9 biology have been demonstrated (Windbichler et al, 2011; Akbari et al, 2013; Gantz et al, 2015; Hammond et al, 2015). A Cas9-based system in *An. stephensi* with a homology-directed repair conversion frequency of ≥0.97 per gamete (Gantz et al, 2015) was at least two orders of magnitude more efficient than what we observed here. The feasibility of using a gene-drive technology for population level impact depends on developing and testing new genetic technologies in mosquitoes, and is supported by a diverse set of techniques for genetic manipulation. The successful design of an active, albeit low frequency, transposon-based synthetic autonomous gene-drive element, affords an additional option to approach population modification.

## Supplementary Material

1

2

## Abbreviations

ECFP: Enhanced cyan fluorescent protein
EGFP: Enhanced green fluorescent protein
DsRed: *Discosoma* sp. red fluorescent protein
G_x_: Post-injection generation number x
pBac: *piggyBac*
