# Supplementary material for: nanos-Driven expression of piggyBac transposase induces mobilization of a synthetic autonomous transposon in the malaria vector mosquito, Anopheles stephensi
Source: Insect Biochem Mol Biol. Author manuscript; Available in PMC 2018 Aug 1. (PMC5580807; doi:10.1016/j.ibmb.2017.06.014)
Supplement: 1 [file NIHMS890827-supplement-1.pptx]

## Slide 1
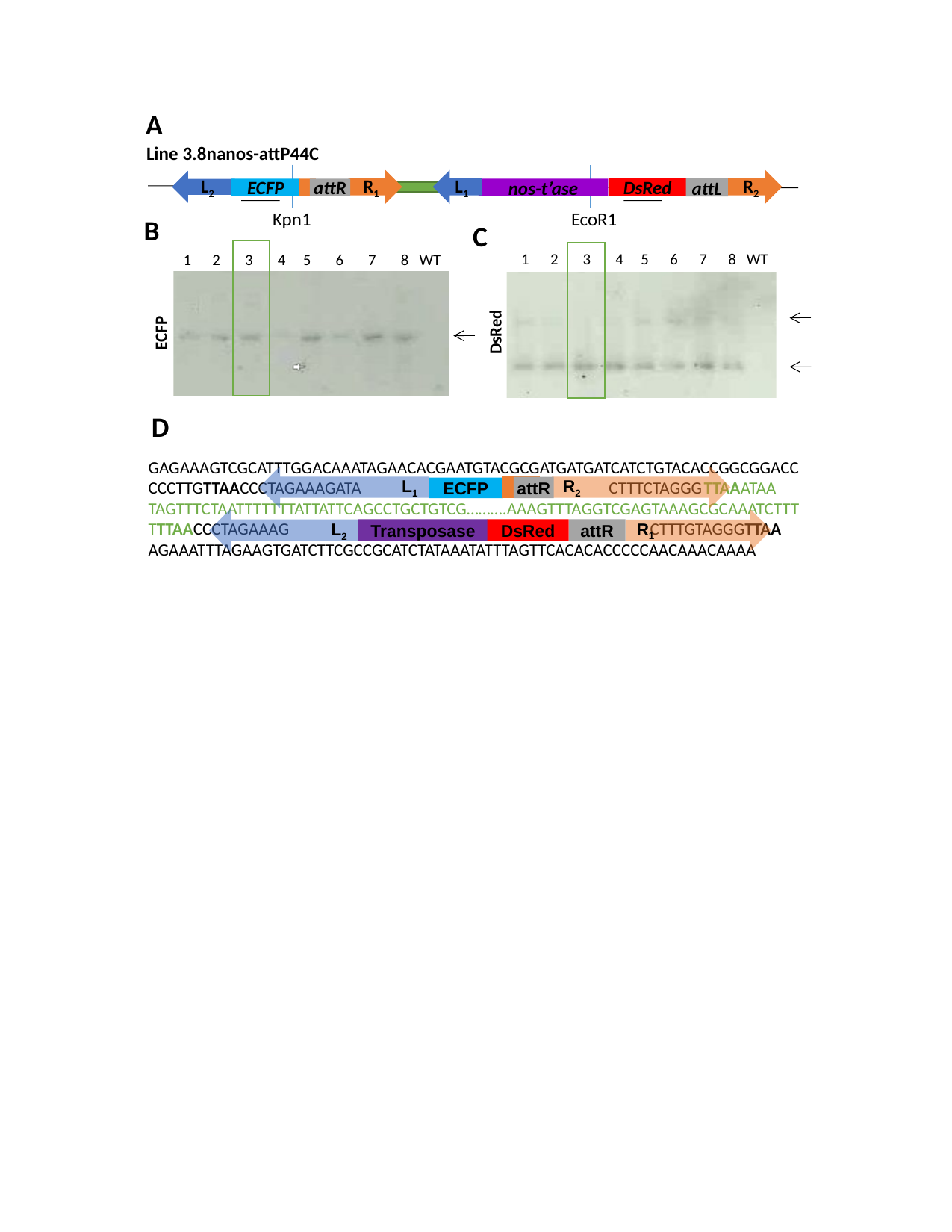

A
Line 3.8nanos-attP44C
L1
R1
R2
L2
attL
ECFP
attR
nos-t’ase
DsRed
Kpn1
EcoR1
B
C
 1 2 3 4 5 6 7 8 WT
 1 2 3 4 5 6 7 8 WT
DsRed
ECFP
D
GAGAAAGTCGCATTTGGACAAATAGAACACGAATGTACGCGATGATGATCATCTGTACACCGGCGGACCCCCTTGTTAACCCTAGAAAGATA A CTTTCTAGGGTTAAATAA
TAGTTTCTAATTTTTTTATTATTCAGCCTGCTGTCG……….AAAGTTTAGGTCGAGTAAAGCGCAAATCTTTTTTAACCCTAGAAAG CTTTGTAGGGTTAA
AGAAATTTAGAAGTGATCTTCGCCGCATCTATAAATATTTAGTTCACACACCCCCAACAAACAAAA
L1
R2
attR
ECFP
L2
R1
Transposase
DsRed
attR
