# Supplementary material for: nanos-Driven expression of piggyBac transposase induces mobilization of a synthetic autonomous transposon in the malaria vector mosquito, Anopheles stephensi
Source: Insect Biochem Mol Biol. Author manuscript; Available in PMC 2018 Aug 1. (PMC5580807; doi:10.1016/j.ibmb.2017.06.014)
Supplement: 2 [file NIHMS890827-supplement-2.docx]

| **Supplemental Table 1. Oligonucleotide primers used in the study.^1^** | | |
| --- | --- | --- |
| **Cloning Primers** | | |
| 0.9nanos 5'UTR | 0.9PRO5U H3 For: AGCTAAGCTTGTGAAAGAACGTGCGACG | |
|  | PRO 5UX1 Rev: AGCTTCTAGACTTTGTGTGGCAAAGAGGG | |
| Nanos 3'UTR | 3UTRBaH1 For: AGCTGGATCCGAGGCAGCATCGTTACGAC | |
|  | 3UTR EcR1 Rev: AGCTGAATTCTCTCGGTCGCTCGGTCGC | |
| piggyBac ORF | pBac XbF: AGCTTCTAGAATGGGTAGTTCTTTAGAC | |
|  | pBacORFBmR: AGCTGGATCCTCAGTCAGAAACAACTTT | |
| attB site amplification | attB FOR: CTGCAGATACAGTGAGCGCGCGTAATACGACTCACTATAGG | |
|  | attB REV: CTGCAGAATTAGATCCCCGGGCGAGCTCGAATTAACCATTGTGGGAACCTCGAG | |
| **RT-PCR** | | |
| piggyBac transposase | pBacaseORF For: ATGGGTAGTTCTTTAGACGATGA | |
|  | pBacORFR456: ACGTTTCAATGATATCTCAGCA | |
| Nanos | For: ATGGCAGAAAGTCGATGTAAAACCAAACAG | |
|  | 5’Race-4Rev: GGCCATTCGCGCCAATTGTCTCTTGTACC | |
| 26S | Anst26S RIB For: AATCCTTCCCCAAGGACATGAACCG | |
|  | Anst26S RIB Rev: TACGAAACAAATCCCATCCTAATCGAAGC | |
| 18S | 18s FOR: GTCTCAAAGGTTAAGCCATGCATGTCTAAG | |
|  | 18sREV: CGGAATCGAACCCTGATTCCCCGTTACCCG | |
| **Probes for Southern blot templates** | | |
| ECFP | CFP For: CTTGTACAGCTCGTCCATGC | CFP Rev: ACGTAAACGGCCACAAGTTC |
| DsRed | DsRed For: CCACCAATGGTGCGCTCCTCC | DsRed Rev:ACGTACACCTTGGAGCCGTA |
| **Characterization of *piggyBac* construct location** | |  |
| Primer set |  |  |
| A | 44C For: TTGATCCCCAATTCTGACAC | 44C Rev: TCCCAGGATATTCCCATTTG |
| B | 44C For: TTGATCCCCAATTCTGACAC | piggyBac5REV: TGACACTTACCGCATTGACA |
| C | CFP For: CTTGTACAGCTCGTCCATGC | CFP Rev: ACGTAAACGGCCACAAGTTC |
| D | Plasm2 For: GGTCGAGTAAAGCGCAAATC | piggyBac5REV: TGACACTTACCGCATTGACA |
| E | DsRed New F: CGCTCCTCCAAGAACGTCAT | DsRed New R: TAGTCCTCGTTGTGGGAGGT |
| **Inverse PCR** | **Primer** | **Reference** |
| piggyBac5FOR | TCTTGACCTTGCCACAGAGG | Handler 1998 |
| piggyBac5REV | TGACACTTACCGCATTGACA | Handler 1998 |
| piggyBac3FOR | CATTTGCCTTTCGCCTTATTTTAGA | Nimmo *et al*. 2006 |
| piggyBac3REV | AAACCTCGATATACAGACCGATAAAAACAC | Isaacs *et al*. 2012 |
| ^1^ All sequences listed in their 5'-3' orientation. | | |
